# Supplementary material for: Assessing COVID-19 pandemic excess deaths in Brazil: Years 2020 and 2021
Source: PLoS One. 2023 May 25;18(5):e0272752. doi: 10.1371/journal.pone.0272752 (PMC10212149; doi:10.1371/journal.pone.0272752)
Supplement: S1 Table — (PDF) [file pone.0272752.s003.pdf]

Table S1. Parameter estimates, standard errors (SE) and random effect predictions for the year 2020 (RE), for the LMMs referring to the fittings in Figs 2-3 and Table 2 of the paper

	All-cause			Cardiovascular			Other Diseases			Neoplasms		
Par	Est	SE	RE	Est	SE	RE	Est	SE	RE	Est	SE	RE
β_0	24539.37	182.15	8.95	6842.59	62.34	2.41	5284.57	53.54	0.00	3947.48	14.19	0.01
β_{11}	-1170.28	135.93	20.75	-459.28	40.43	6.68	-265.42	36.15	0.00	78.76	13.59	1.01
β_{21}	-859.39	110.3	0.73	-334.13	31.06	-0.16	-157.01	36.43	0.79	-55.41	12.16	-1.23
β_{12}										-26.74	9.87	
β_{22}										2.81	9.83	
β_3	6.79	1.15		1.04	0.39		3.09	0.34		2.35	0.09	
ω	0.16			0.15			0.17			0.12		
σ_b	116.55			53.73			0.19			0.94		
σ_{b11}	204.11			62.64			0.24			21.10		
σ_{b21}	83.16			13.40			21.40			16.53		
σ_{b12}												
σ_{b22}												
σ	600.54			194.46			194.77			95.02		
ϕ	0.64			0.54			0.69			0.19		
	Respiratory			External†			Ill-defined			Other Infect.		
Par	Est	SE	RE	Est	SE	RE	Est	SE	RE	Est	SE	RE
β_0	2961.94	44.52	0.04	2824.67	50.02	-58.61	1302.64	49.31	-63.21	1069.37	8.88	8.14
β_{11}	-310.81	29.76	-0.11	-34.83	33.12		-80.46	7.07		46.68	12.27	9.89
β_{21}	-385.6	29.91	0.09	221.35	52.09	-10.19	56.44	10.18	-3.04	-36.5	6.03	1.27
β_{12}	26.25	24.63		59.46	63.87	64.00	-38.48	8.68	-6.02	4.54	6.04	1.40
β_{22}	46.41	23.16		-			-2.4	6.54		7.00	7.13	-0.78
β_3	0.88	0.28		-			0.77	0.25				
ω	0.15			-			0.21			0.12		
σ_b	9.52			115.89			75.08			19.50		
σ_{b11}	5.66									26.60		
σ_{b21}	5.55			102.30			16.77			9.73		
σ_{b12}				98.28			12.50			9.72		
σ_{b22}										13.27		
σ	149.38			108.20			59.89			39.77		
ϕ	0.73			0.17			0.27			0.22		

†: The coefficient estimates for External causes refer to the parameters of the third-order polynomial since FS did not provided an adequate fit to the data.
