# Supplementary material for: Assessing COVID-19 pandemic excess deaths in Brazil: Years 2020 and 2021
Source: PLoS One. 2023 May 25;18(5):e0272752. doi: 10.1371/journal.pone.0272752 (PMC10212149; doi:10.1371/journal.pone.0272752)
Supplement: S3 Table — (PDF) [file pone.0272752.s005.pdf]

Table S3. Parameter estimates, standard errors (SE) and random effect predictions for the year 2020 (RE), for the LMMs referring to the fittings in Figs 5-6 and Table 3 of the paper

	Male			Female			Race/Color: White			Race/Color: Brown		
Par	Est	SE	RE	Est	SE	RE	Est	SE	RE	Est	SE	RE
β_0	13831.89	120.58	18.92	10682.42	78.04	0.85	12510.27	102.67	-9.67	8961.15	114.68	35.98
β_{11}	-545.79	81.85	47.44	-548.32	62.83	2.50	-883.22	70.30	-14.31	-165.63	58.40	23.45
β_{21}	-457.25	51.18	-0.17	-503.96	61.19	0.24	-512.36	78.70	-9.30	-304.32	41.07	-0.04
β_{12}	60.42	46.69		89.90	42.71		126.34	51.80				
β_{22}	-30.58	45.61		-3.45	41.54	-0.03	-41.57	51.07	0.29			
β_{13}	27.27	41.60										
β_{23}	112.85	40.13										
β_3	2.62	0.74		4.09	0.50		3.31	0.65		3.81	0.69	
ω	0.16			0.16			0.16			0.16		
σ_b	119.09			48.97			77.14			120.91		
σ_{b11}	151.81			99.15			99.22			96.95		
σ_{b21}	14.74			89.45			124.25			6.62		
σ_{b22}				11.48			19.82					
σ	319.40			276.05			357.85			233.75		
ϕ	0.52			0.59			0.53			0.64		
	Race/Color: Black			Race/Color: Asian			Race/Color: Indigenous			Age: 0-19		
Par	Est	SE	RE	Est	SE	RE	Est	SE	RE	Est	SE	RE
β_0	1786.20	11.01	0.01	137.57	1.98	0.28	69.25	1.55	0.32	1435.35	21.10	-21.77
β_{11}	-93.53	10.42	3.66	-7.77	1.07		1.66	0.79		8.82	-5.91	
β_{21}	-38.91	9.32	2.73	-4.17	1.76		-4.28	0.79		-6.76	9.04	-11.31
β_{12}												
β_{22}												
β_3	1.22	0.07		9.46	3.37		15.73	2.62		-0.75	0.12	
ω	0.17			0.16			0.15			0.12		
σ_b	0.60			1.48			1.31			25.02		
σ_{b11}	16.30									17.88		
σ_{b21}	11.99			3.16						18.73		
σ_{b12}				14.59								
σ	62.18			12.22			8.91			43.81		
ϕ	0.34			-			-			-		
	Age: 20-39			Age: 40-59			Age: 60-79			Age: 80+		
Par	Est	SE	RE	Est	SE	RE	Est	SE	RE			
β_0	2355.69	43.90	34.77	4787.70	54.79	47.18	8965.41	64.52	-0.31	6947.33	75.56	-5.97
β_{11}	13.24	13.83		-137.06	21.47	5,27	-472.89	58.67	-3.72	-570.95	48.49	-3.39
β_{21}	7.54	13.56		-93.06	27.27	21.07	-438.31	37.41	-0.03	-416.27	41.21	-0.08
β_{12}							66.85	33.96		68.31	37.40	
β_{22}							0.71	32.48	-0.01	-23.45	46.27	-2.17
β_3	-0.91	0.26		-0.38	0.33		4.54	0.41		4.12	0.47	
ω	0.26			0.17			0.16			0.15		
σ_b	48.42			60.13			45.28			58.78		
σ_{b11}				25.46			8.82			61.81		
σ_{b21}				47.51						6.69		
σ_{b12}							5.50					
σ_{b22}										69.54		
σ	96.37			121.96			228.99			245.32		
ϕ	0.52			0.51			0.55			0.58		
