# Supplementary material for: Assessing COVID-19 pandemic excess deaths in Brazil: Years 2020 and 2021
Source: PLoS One. 2023 May 25;18(5):e0272752. doi: 10.1371/journal.pone.0272752 (PMC10212149; doi:10.1371/journal.pone.0272752)
Supplement: Attachment — Submitted filename: Response to Reviewers.pdf [file pone.0272752.s017.pdf]

Luzia Aparecida Trinca
Institute of Biosciences, Unesp, Botucatu, São Paulo, Brazil
+55 14 997895960
luzia.trinca@unesp.br

To
Dr Queiroz - Academic Editor, PLOS ONE

December 15, 2022

Thank you for your decision letter of September 14, 2022, and for the opportunity to revise our manuscript. My co-authors and I are pleased to submit our revised manuscript titled "Assessing COVID-19 pandemic excess deaths in Brazil: years 2020 and 2021" (Ref: PONE-D-22-20908) for your reconsideration for publication in PLOS ONE.
We thank you and the reviewers for your time and effort in reviewing our manuscript. The feedback has been invaluable in improving the content and presentation of the paper. We have revised our manuscript according to all of your and the reviewers' comments. We highlight that while revising the manuscript, we were aware of significant updates to the data set we used. Given the concerns raised by the reviewers, we took advantage of more reliable data and performed all the analysis again. The changes, which we recognize, are very substantial, are highlighted in the attached appropriate file.

Responses to the Editor

1. Test alternative models, such as GEE, and discuss the differences in the results

We have tested a GEE approach and included some minimal comparisons. The GEE results show a tendency to underestimate expected deaths, but our great concern is accounting for the uncertainties in the forecasts. We do not raise this element in the paper because it is already a long paper, and we felt the issue requires much deeper treatment, out of the aims of our paper. We extend this point a bit more in the responses for the reviewers.

2. Focus on the modeling strategy instead. Perhaps you could explain it more deeply, instead of explaining the regression model

Done. We have removed technical details from the paper and transferred the LMM theory, estimation of excess deaths, etc... to the Support Information.

3. Copyediting - some parts of the paper are a little bit confusing

We tried to be more direct.

4. The mathematical notation is very confusing

Simplifications were applied.

5. Further discuss expected delay in the reporting mortality 2021 data

We discussed the issue and have also included details based on preliminary and final 2020 data, so that some guesses of possible impacts of delay for 2021 can be made.

6. Introduction should brief and specific description on Brazilian epidemic context

Done.

7. Information on missing data to the important covariates such as race/color are unavailable. In which direction this point may have raised interpretative distortions?

Done. We investigated the missing patterns and tried tracking their source. Based on that, we included, in the discussion, our view of the biases direction.

8. Add the key aspects on the study limitations and strong point

Done.

Responses to the Reviewers

1. Is the manuscript technically sound, and do the data support the conclusions?

Reviewer #1: Yes

Reviewer #2: Yes

2. Has the statistical analysis been performed appropriately and rigorously?

Reviewer #1: I Don't Know

There are many alternatives for obtaining baseline forecasts during pandemic periods. The main differences between them are related to the amount of information required and the model assumptions. Our approach requires mortality data only from as large as the wanted historical death period. The model involves classical assumptions while considering correlation and heterogeneity. The biggest impact, we believe, is the explicit accounting for all uncertainties in forecasting deaths for the non-observed period. It is our opinion that quite a few methods being used do not account for all uncertainty, resulting in unreliable prediction precision.

Reviewer #2: Yes

3. Have the authors made all of the data underlying the findings in their manuscript fully available?

Reviewer #1: Yes

Reviewer #2: No

The data is publicly available online from the pages of the Health Ministry, Brazilian Government and we have fully referenced the site.

4. Is the manuscript presented in an intelligible fashion and written in standard English?

Reviewer #1: Yes

Reviewer #2: No

We rewrote large parts and revised the whole text. We hope we improved this point.

5. Review Comments to the Author

Reviewer #1:

From a general point view, this is an interesting and well written paper. Even so, I am not all convinced that the estimates are sufficiently reliably due to the expected delay in the reporting mortality 2021 data in Brazil, even so the authors may wish to consider the following comments.

During the revision process, SIM data were updated. We performed all the analyses again and, in this revised version, present results for the updated data. Still, 2021 is not final. We discuss, in the manuscript, the expected impacts of the reporting delay of mortality for 2021.

Overall, the introduction section would benefit from a brief and specific description on Brazilian epidemic context, mainly scientific denialism and its consequences in public health terms, such as in regard to the avoidable deaths and its relationship with excess deaths.

We rewrote the introduction and implemented the suggestion.

In the following introduction sentence "By the end of 2021, Brazil's coronavirus disease (COVID-19) death toll was 619 334 (11.4% of the world), putting the country among the most affected, behind the USA only", instead overall mortality in December/2021 would be useful changing for the overall mortality in the end of August/2022.

Done.

Details on the excess deaths approach would be better suited in the methods (or discussion) section because there are already a large amount studies on the topic both national and international Covid-19 pandemic literature. Instead, the authors may address to the readers highlighting the pandemic impact on the mortality profile around the world and/or Brazil and adding one or more hypothesis into introduction section in regard expected results to the general or specific mortality causes of death.

We addressed these suggestions.

In "Materials and methods" section, specifically in the following sentence "We obtained the publicly available data (2015-2021) from the Mortality Information 58 System (SIM) [22], Ministry of Health, Brazil Government, on 7 March 2022" it is not sufficiently clear if the 2020 mortality data are

officially considered finalized. Whereas the 2021 data were made available preliminarily is crucial updating the data set with the last version (June 2022).

Done.

In the results section the information on missing data to the important covariates such as race/color are unavailable. In which direction this point may have raised interpretative distortions?

Done.

There is a clear unbalance between results and discussion section, showing that substantial data portion not was discussed by the authors.

Finally, is lacking to add the key aspects on the study limitations and strong points. For example, delay deaths notification, underreporting in poorest Brazilian regions, changes in the coding of underlying causes and the dates of deaths, impact of the different modeling approaches to estimate excess deaths, hard interpretation of the disaggregated analyses to the youngest people or also evaluating data on excess deaths in different pandemic stages.

We addressed these points, although we believe just touching the impact of different estimation methods. That would be quite a deep study, we think.

Reviewer #2: Major changes

1. About the model, the author says that one criticism of LMM is the assumption of normality which is solved using weekly data. Why do not use Generalized Estimating Equations instead? The GEE models do not assume normality of data. Which could be the main differences in the results?

We have included a brief comparison of LMM and a GEE approach.

2. I strongly recommend eliminating all the explanation of LMM. Such models are very well known, and the aim of the paper is not to develop a new statistical procedure but to apply it. The authors could focus on the modeling strategy instead. Perhaps you could explain it more deeply.

Done.

3. The rest of the mathematical description of the model could be relocated at the Appendix. It is unnecessary to explain it with this detail because, as I stated above, it is not a paper on a new methodological development.

We tried to present minimal mathematical details in the paper and allocated the details in the Supporting Information.

4. Check in the literature if “Brown” is a category of race and justify it.

Brown ("Parda" in Portuguese) is a category of multidimensional criteria supposed to collect

ethnic-racial information, included in official statistics in Brazil. The question is "Sua cor ou raça é:" (Your color or race is:). The available categories are "Branca" (White), "Preta" (Black), "Parda" (Brown), "Amarela" (Asian) and "Indigena" (Indigenous). The interpretation of any answer should be "as one perceives oneself" in the context of the Brazilian population. In the case of mortality data, a family member answers the question, if possible. So, even if we accept that race exists, neither "White" is a category of race in our data.

5. Race is not the same as skin color according with recent literature. Please review the proper literature about it, for instance:

Jablonski, NG. Skin color and race. Am J Phys Anthropol. 2021; 175: 437– 447. https://doi.org/10.1002/ajpa.24200

Thank you for bringing to attention this rather complex theme. This point is the one we felt the most difficult to address and we must admit the discussion is out of our expertise. What we can explain is that we used race/color (we opt now to remove "skin" because it is not as it was asked as detailed above) to account for a multi-dimensional factor that diversifies the Brazilian population, in a social context instead of a biological marker. It is the only information available in the mortality data that allows exploration of the pandemic impact in groups with different hardships, which perhaps is linked to the inhuman past that we cannot erase or pretend did not happen. In Brazil, the information on this factor is considered crucial to generate subsidies and indicators that favor decision-making policies (governmental and non-governmental) for resource allocation, and implementation of programs to reduce inequalities in opportunities in general, including healthcare. Are there better approaches? We truly have no idea for this country. Meanwhile, we hope that the stratified analysis we present can show, at least, useful information for improving transparency of the pandemic's impacts on our most diverse population. We tried to add some context for this factor in the paper.

Minor changes

1. Copyediting

Done.

2. I recommend to review and include following references:

Lima, E.E.C., Vilela, E.A., Peralta, A. et al. Investigating regional excess mortality during 2020 COVID-19 pandemic in selected Latin American countries. Genus 77, 30 (2021). https://doi.org/10.1186/s41118-021-00139-1

L B Nucci, C C Enes, F R Ferraz, I V da Silva, A E M Rinaldi, W L Conde, Excess mortality associated with COVID-19 in Brazil: 2020–2021, Journal of Public Health, 2021

Done. Thanks for pointing these to us.

3. I strongly recommend to review this paper:

Palacio-Mejía, L. et al. (2022). Leading causes of excess mortality in Mexico during the COVID-19 pandemic 2020–2021: A death certificates study in a middle-income country, The Lancet Regional Health – Americas,

https://doi.org/10.1016/j.lana.2022.100303

Done.

4. How the results and methods of this paper are different from

https://ourworldindata.org/excess-mortality-covid ?

As we understand, ourworldindata.org presents results from a few methods, for example, Karlinsky and Koback (2021) and the so-called WHO method. The first method estimates the baseline using a regression model with smoothing-spline-based weekly effects (normality assumed). The second one uses a GLM (Generalized Linear Model), with Negative Binomial or Poisson distributions. Both methods do not consider the correlation among death counts over time. We believe the different methods impact mainly the precision of forecasts. For the first method, ourworldindata.org does not present any uncertainty measure, and point forecasts are comparable to ours. The WHO method calculates precision for the forecasts based on simulations after fitting the model. It needs those simulations to really incorporate the uncertainty with respect to future deaths because GLMs are not devised for predictions. We briefly mention this in the paper. The same issue arises for GEE. As we understand, GEEs are devised for estimating a mean profile, not for predicting a new observation. We can project the estimated equation to a future time and get a point prediction but the precision for that prediction is not reliable if we account only for the error in the parameter estimates. In contrast, LMM allows explicit accounting of uncertainties around point forecasts in a simpler way, just extending linear model theory formulae, no simulations or unusual approximations are required. That is because of the additive nature of random terms in LMMs. Details about this property were transferred to the Support Information in this revised version.

5. The quotation is quite confusing.

Sorry, we had difficulty interpreting this. For reference citations, we used the journal style and revised some specific points we had been using authors' names to address the work.

6. Define the acronyms of the states at the Appendix or perhaps the would be included with the maps.

We have substituted the maps for more informative graphs. Now the states' full names are displayed.

7. The mathematical notation is very confusing, and in some parts the variables, functions and rank notation are not defined. I strongly recommend to check it and simplify it.

Done.

All authors have read and approved the changes made to the manuscript. We hope that the revised paper is now suitable for inclusion in PLOS ONE and we look forward to hearing from you.

Your Sincerely,

Luzia Aparecida Trinca
