# Supplementary material for: Assessing COVID-19 pandemic excess deaths in Brazil: Years 2020 and 2021
Source: PLoS One. 2023 May 25;18(5):e0272752. doi: 10.1371/journal.pone.0272752 (PMC10212149; doi:10.1371/journal.pone.0272752)
Supplement: Attachment — Submitted filename: Response to Reviewers.pdf [file pone.0272752.s018.pdf]

Response to the Editor and Reviewers

Responses to the Editor

Thank you for your decision letter of February 15, 2023, and for the opportunity to revise our manuscript. My co-authors and I are pleased to submit our revised manuscript titled "Assessing COVID-19 pandemic excess deaths in Brazil: years 2020 and 2021" (Ref: PONE-D-22-20908R1) for your reconsideration for publication in PLOS ONE.

We thank you and the reviewers for your time and effort in reviewing our manuscript. The feedback has been invaluable in improving the content and presentation of the paper. We have revised our manuscript according to all of your and the reviewers' comments.

In addition to our responses to specific points below, we would like to inform we have checked the reference list and:

1. We have corrected some issues, mainly with respect to doi specifications.
2. We have added one reference, currently number 43 because it supports our answer to some points asked by Reviewer # 3.
3. Furthermore, we removed reference number 45 from previous version and, instead, cited the data link on the web in the text (line 278).
4. We are not aware of any retracted work in our reference list.

Additional Editor Comments:

The authors may incorporate the comments suggested by Reviewers 3 and 4. Secondly, it is suggested to add conclusion section along with limitations and future recommendations.
We have included a conclusion section.

Responses to Reviewers

Reviewer's Responses to Questions

2. Is the manuscript technically sound, and do the data support the conclusions?

The manuscript must describe a technically sound piece of scientific research with data that supports the conclusions. Experiments must have been conducted rigorously, with appropriate controls, replication, and sample sizes. The conclusions must be drawn appropriately based on the data presented.

Reviewer #1: Yes

Reviewer #3: Partly

Our comment: We have included a web link for the aggregated data and some program code for the analyses and a conclusion section in the main text, and tables with model parameter estimates in the supplementary information.

Reviewer #4: Yes

3. Has the statistical analysis been performed appropriately and rigorously?

Reviewer #1: Yes

Reviewer #3: No

Our comment: We have performed diagnostics for the models fitted and did not find any serious violations on models' assumptions indicating the models are reasonable approximations for the data. We opted to show just some of the diagnostic graphs in the Supplementary Information material because of the large number of figures.

Reviewer #4: Yes

4. Have the authors made all data underlying the findings in their manuscript fully available?

Reviewer #1: Yes

Reviewer #3: No

Our comment: Data is publicly available, online, from the pages of the Health Ministry, Brazilian Government, as we had fully referenced in the References section. In this revision, we have included the links in the text, subsection Data (lines 53/54) and also, our github repository with processed (aggregated) data, program code, etc (line 82).

Reviewer #4: Yes

5. Is the manuscript presented in an intelligible fashion and written in standard English?

Reviewer #1: Yes

Reviewer #3: Yes

Reviewer #4: Yes

6. Review Comments to the Author

Reviewer #1: After careful review, I feel the revised version clarifies important points, mainly regarding the Materials and methods section, turning up the manuscript results even more robust. I am therefore in favour of the accept this new manuscript version.
Thank you for your careful review and comments in the both versions which helped us to improve our work.

Reviewer #3: The authors incorporated most of the concerns raised by the reviewers. However, still, some of the work is not incorporated. i.e.
(1) There is still irrelevant statements in the manuscript, for instance, "An advantage of the linear mixed model is the flexibility to capture year-trend while dealing with the correlations among death counts over time".
Thank you for your careful review. We have, hopefully, removed all the irrelevant parts in the text.

(2) There is other methods available in the literature as mentioned by the 2nd reviewer. Why the authors focused on LMM only besides its flexibility and relationship among the variables?
Yes, there are many different methods available and, initially, based on Verbeeck et al. (2021) we chose LMM. These authors pointed out some drawbacks of several methods and favored the LMM. However, based on the comments of Reviewer 2 in the first version, we did some comparisons between three methods, one of them being the method mentioned by Reviewer 2, the GEE. In Table 1 of the paper, we show that LMM and GEE perform similarly in terms of point predictions while the five-year average is much poorer. In Figure 1 we compare the fitted curves from LMM and GEE where we can see LMM captures better the peaks. Allied with that, as explained in the text, GEE is not suited for future predictions (and we want to predict death counts had the pandemic not occurred). That is because the method does not consider errors associated with future points in time, resulting in practically null standard errors for the predictions. In our opinion, that can mislead conclusion on excess deaths. Another method that would be sound is GLMM but it requires simulations to properly account for the uncertainty around predictions. Based on that, we chose to use LMM which, explicitly, incorporates all uncertainty around predictions. Details on standard errors calculations are presented in S1 File.

(3) The authors only displayed the tables for expected and estimated deaths, why not they showing the coefficients of LMM?
New tables with all the parameter estimates are provided in the Supplemental Information, S1 Tab and S3 Tab.

(4) Why the authors included the GLM and AR in the discussion section? is there really need to discuss it in the discussion section?
We have removed some details of these methods in the discussion

but found it informative to keep their names because the five-year average tends to overestimate excess. Besides, the completest previous work on excess deaths in Brazil (reference [6]) used GLM, and we felt it would be flawed not to include it in the discussion.

(5) The authors showed that the South is less impacted while the North and Central-West had the highest in Brazil. It is suggested that also bring the possible and general reasons/factors.

We added some possible explanations for the heterogeneity across the country. We have included, in Figure 4 of the paper, the Human Development Index and vaccination coverage as surrogates for life quality, education, economical and health capacity of each state in an attempt to draw some possible explanations for the heterogeneous impact. However, it is not possible, with our data and analysis, to really account for the factors behind the heterogeneous impact across the country.

(6) It is suggested that to add conclusion section along with limitation and future recommendation.

The Conclusion section is included.

(7) Overall the paper need technical improvement, i.e. to remove irrelevant materials.

Done.

Reviewer #4: The authors analyzed COVID-19 pandemic-induced mortality in Brazil over a period of two years. Although, the paper lacks novelty but strongly addresses the epidemiological patterns in this study. The comments of previous reviewers are addressed appropriately. I recommend adding a few lines about the implications of the study for any future pandemics like COVID-19.

Thank you for your careful review. We included a conclusion section.

There are a few editorial mistakes e.g.,

1. in line 253 add "and we found.."

Fixed.

2. Line 85 starts with reference number 18. Please change it to author according to the refereeing guidelines.

Done.

3. I suggest changing Line 106, "Another possible approach is the application of the Generalized Estimation 105 Equations (GEE) formulation [32], which, while considering correlations among the 106 observations, does not require any distributional assumption" to "Another possible approach is the application of the Generalized Estimation 105 Equations (GEE) formulation [32], which does not require any distributional assumption when correlations among the 106 observations considered"

Thanks for the suggestion. In order to express the proper meaning we have rephrased it to "Another possible approach is the applica-

tion of the Generalized Estimation Equations (GEE) formulation [32], which does not require any distributional assumption and allows correlations among the observations."
