# Supplementary material for: Assessing COVID-19 pandemic excess deaths in Brazil: Years 2020 and 2021
Source: PLoS One. 2023 May 25;18(5):e0272752. doi: 10.1371/journal.pone.0272752 (PMC10212149; doi:10.1371/journal.pone.0272752)
Supplement: Attachment — Submitted filename: Response to Reviewers.pdf [file pone.0272752.s019.pdf]

Response to the Editor and Reviewers

Responses to the Editor

Thank you for your decision letter of April 24, 2023, and for the opportunity to revise our manuscript. My co-authors and I are pleased to submit our revised manuscript titled “Assessing COVID-19 pandemic excess deaths in Brazil: years 2020 and 2021” (Ref: PONE-D-22-20908R2) for your reconsideration for publication in PLOS ONE.

We thank you and the reviewers for your time and effort in reviewing our manuscript. The feedback has been invaluable in improving the content and presentation of the paper. We have revised our manuscript according to all of your and the reviewers’ comments.

In addition to our responses to specific points below, we would like to inform we have checked the reference list and:

1. We have added a few references accordingly to our revision.
2. We are not aware of any retracted work in our reference list.

Additional Editor Comments:

Minor changes
1) Abstract
From ”... from the Health Ministry, the Brazilian Government, to fit linear mixed models for forecasting ... To ”... from the Brazilian Health Ministry for forecasting....”
Done.

2) Introduction – pg 2, line 10
There is no delegation of the Supreme Court for states and municipalities. Supreme Court has decided that local governments should opt for more severe NPIs than the Federal Government. See ”Abrucio, F. L., Grin, E., & Segatto, C. I. (2021a). Brazilian Federalism in the Pandemic. In Peters, G., Abrucio, F. & Grin, E (ed). American Federal Systems and COVID-19. Emerald Publishing Limited” for a full description of the Supreme Court’s role during the pandemic.
Thank you for clarifying this issue to us. We have rewritten the text and included references.

3) Introduction – pg 2, line 23 From "…the huge…" To "… huge…"
Done.

4) Methods – pg 5, line 106 From ". Yet, as far we understand, it .." To "Yet, it.."
Done.

5) Methods – pg 5, line 114 From ". Its drawback, in our opinion, is .." To "Its drawback is." The affirmation should be based on a literature reference on the methodology.
Done. We revised this part and added references.

6) All links should be changed for References. The links should be presented in the Reference list.
Done. Unfortunately, in the previous revision, we decided to move the links to the text because a referee kept insisting we did not provide access to the data, although the links were in the reference list. Now, in this version, the links are back to the Reference list. We hope we got it right this time.

7) Estimated the effect of Asian and Indigenous groups separated. I understand that they are both small groups, but the effects of COVID-19 on these two groups are different. Asians have higher social status and Indigenous groups were heavily affected by government policies during the pandemic.
Done. Thank you for the suggestion. The dis-aggregated analysis show an unexpected pattern for Asians and we added plots of deaths by cause of death, for each group to try understanding the patterns. Those graphs are in the Supporting Information.

8) The text should be rewrite whenever numbers is used in the place of reference Names. For example Pg. 22, line 502-503 From "… also pointed out in [19], [26] and [44], although [26] and [44] used different deaths-cause grouping…." To "…also pointed out in other studies [19, 26, 44], although some of then [26, 44] used differen deaths-cause grouping…"
Done.

Responses to Reviewers
Reviewer's Responses to Questions

***The Reviewers responses for Itens 1 to 5* were "Yes", so thank them for recognizing the improvements in our manuscript.**

6. Review Comments to the Author

Please use the space provided to explain your answers to the questions above. You may also include additional comments for the author, including concerns about dual publication, research ethics, or publication ethics. (Please upload your review as an attachment if it exceeds 20,000 characters)

Reviewer #3: The authors incorporated most of the concerns raised the reviewers. However, limitations and future recommendations are still not provide. Please provide these well.

In this version, we have described the strengths and limitations of our work in a subsection within the Discussion. Some recommendations are outlined in the Conclusions.

Reviewer #4: I have no further comments on the manuscript. The authors have addressed all the comments appropriately.

Thank you!
